# 1-Palmitoyl-2-Linoleoyl-3-Acetyl-rac-Glycerol (PLAG) Rapidly Resolves LPS-Induced Acute Lung Injury Through the Effective Control of Neutrophil Recruitment

**DOI:** 10.3389/fimmu.2019.02177

**Published:** 2019-09-18

**Authors:** Ha-Reum Lee, Su-Hyun Shin, Joo Heon Kim, Ki-Young Sohn, Sun Young Yoon, Jae Wha Kim

**Affiliations:** ^1^ENZYCHEM Lifesciences, Seoul, South Korea; ^2^Division of Systems Biology and Bioengineering, Cell Factory Research Center, Korea Research Institute of Bioscience and Biotechnology, Daejeon, South Korea; ^3^Department of Functional Genomics, University of Science and Technology, Daejeon, South Korea; ^4^Department of Pathology, EulJi University School of Medicine, Daejeon, South Korea

**Keywords:** acute lung injury, neutrophil, transmigration, inflammation, PLAG, resolution

## Abstract

Acute lung injury (ALI) is an acute respiratory failure that is associated with excessive neutrophil recruitment and high mortality. To assess the efficacy of 1-palmitoyl-2-linoleoyl-3-acetyl-rac-glycerol (PLAG) as a therapeutic agent for ALI, this compound was administered orally to mice challenged with an intranasal dose of lipopolysaccharide (LPS). Using this model, we found that PLAG promotes resolution of ALI through effective control of LPS-induced neutrophil infiltration, endothelial permeability, and inflammatory chemokine production. In addition, the Toll like Receptor 4 (TLR4) endocytosis/exocytosis cycle was significantly accelerated in Raw 264.7 cells co-treated with PLAG/LPS, as compared to cells treated only with LPS. During this cycle, a PLAG-induced exotoxin clearance pathway was observed to occur through the prompt assembly of nicotinamide adenine dinucleotide phosphate (NADPH) units and production of reactive oxygen species (ROS), which ultimately lead to earlier LPS clearance. We further detected reduced expression, as well as faster return to homeostatic levels, of macrophage inflammatory protein (MIP)-2, in PLAG/LPS- vs. LPS-treated cells. MIP-2 is a main inducer of neutrophil migration that is mainly controlled by interferon regulatory factor 3 (IRF3) activation and is involved in the TLR4 endosomal-signaling pathway. PLAG induced TLR4-mediated TRIF-related adaptor molecules/Toll-interleukin receptor (TIR) domain-containing adaptor protein including interferon (IFN)-β/IRF3 endosomal signaling, leading to rapid association of TRAM/TRIF and TLR4 and earlier IRF3 phosphorylation in PLAG/LPS-treated vs. LPS-treated cells. PLAG specificity was further verified with PLAG analogs and metabolites known to control excessive neutrophil infiltration, suggesting that this acetylated diacylglycerol has a unique biological role in neutrophil motility. Thus, our data indicate that PLAG may represent a potential therapeutic agent for resolution of LPS-induced lung inflammation through effective MIP-2 modulation.

## Introduction

Acute lung injury (ALI) is a severe inflammatory lung disease that is characterized by the disruption of the lung alveolar-capillary membrane barrier. This leads to a massive infiltration of neutrophils into the interstitium and the bronchoalveolar space, as well as an excessive inflammatory response (1). The activation and transmigration of neutrophils are considered an essential step in ALI progression. Neutrophils play a key role in the innate immune system, as these cells are the first leukocytes to migrate to regions of acute inflammation (2). Neutrophils cross the blood vessel endothelium into infected tissue and eliminate invading pathogens via multiple killing mechanisms, including phagocytosis, degranulation, and neutrophil extracellular traps (NETs) (3, 4). Notably, neutrophils secrete numerous cytokines and chemokines that influence other immune cells and are thus key regulators of inflammation (5). Although the transmigration and activation of neutrophils are absolutely essential for infection clearance, excessive recruitment, and aberrant activation of neutrophils leads to severe host tissue damage and may ultimately cause death (6).

Lipopolysaccharide (LPS) is a Gram-negative bacterial toxin recognized by Toll-like receptor 4 (TLR4), which is used to induce both acute and chronic tissue injury in animal models of inflammation (7, 8). When LPS infiltrates into the lung, macrophages are activated by the LPS-receptor complex, TLR4/MD-2, and this stimulates the translocation of nicotinamide adenine dinucleotide phosphate (NADPH) oxidase (NOX) from the cytosol to membrane (9). Activated NOX produces reactive oxygen species (ROS) that are able to directly kill pathogens and stimulate increased expression of interleukin (IL)-8 and macrophage inflammatory protein (MIP)-2 (10). Neutrophil migration is regulated by the response to chemokine gradients, and MIP-2, in particular, is a major chemokine involved in the induction of neutrophil migration (11, 12).

TLR4-associated signaling pathways are classified based on the involvement of two main adaptor proteins, referred to as myeloid differentiation primary response protein 88 (Myd88) and Toll-interleukin receptor (TIR) domain-containing adaptor protein inducing interferon (IFN)-β (TRIF) (13). The Myd88-dependent pathway, which occurs mainly at the plasma membrane, is mediated by Myd88 and the TIR domain-containing adapter protein (TIRAP). This pathway activates nuclear transcription factor kappa B (NF-κB) signaling and leads to induction IL-1β and tumor necrosis factor (TNF) (14). In contrast, the TRIF-dependent pathway utilizes the TRIF-related adaptor molecules (TRAM) and TRIF and is associated with endocytosis of the activated TLR4 receptor. This occurs at the endosomal membrane following TLR4 internalization and activates signaling via the interferon regulatory factor 3 (IRF3) transcription factor, as well as IFN-β secretion (15). Critically, the TLR4-mediated signaling pathways play crucial roles in the immune response and in host defense by acting as sensors of microbial infection.

The acetylated diacylglycerol 1-Palmitoyl-2-linoleoyl-3-acetyl-rac-glycerol (PLAG), is a mono-acetyl-diglyceride that has been isolated from the antlers of sika deer and can be chemically synthesized from glycerol, palmitic acid, and linoleic acid (16). Synthesized PLAG is chemically identical to its naturally isolated form (17). In a previous study, we found that PLAG exerts therapeutic effects when administered in combination pegfilgrastim for treatment of chemotherapy-induced neutropenia. PLAG modulated neutrophil transmigration, and significantly reduced fluorouracil/scratching-induced oral mucositis and cachexia (18, 19). Here, we investigated the effects of PLAG on neutrophil migration and the resolution of inflammation in an LPS-induced mouse model of ALI and on TLR4 signaling of macrophage cells in culture. We show that PLAG modulates TLR4 endocytosis-dependent endosomal signaling, TRAM-TRIF-IRF3-mediated signaling, and ultimately can control excessive neutrophil infiltration through modulation of MIP-2 expression. These findings suggest that PLAG may have therapeutic potential for treatment of ALI and other severe inflammatory diseases.

## Materials and Methods

### LPS-Induced ALI Mouse Model

Balb/c mice (9-week to 11-week-old males) were purchased from Koatech Co. (Pyongtaek, Republic of Korea) and maintained under specific pathogen-free (SPF) conditions. For the ALI model, mice were anesthetized with 2,2,2-Tribromoethanol (150 mg/kg; Sigma-Aldrich, St. Louis, MO, USA) by intraperitoneal injection and administered LPS intranasally (25 mg/kg; Sigma-Aldrich). PLAG (10, 50, or 250 mg/kg, Enzychem Lifesciences Co., Daejeon, Republic of Korea) was administered orally. Collection of bronchoalveolar lavage fluid (BALF) was performed by tracheal cannulation, using cold phosphate-buffered saline (PBS). Complete blood counts (CBCs) were performed using the Mindray BC-5300 Auto Hematology Analyzer (Shenzhen Mindray Bio-medical Electronics, China).

### Ethics Statement

All animal experimental procedures were performed in accordance with the Guide and Use of Laboratory Animals (Institute of Laboratory Animal Resources). All experiments were approved by the Institutional Review Committee for Animal Care and Use of KRIBB (Korea Research Institute of Bioscience and Biotechnology, Daejeon, Republic of Korea), approval number KRIBB-AEC-16031.

### Cell Culture and Primary Neutrophil Isolation

Raw 264.7 (American Type Culture Collection [ATCC], Manassas, VA, USA) cells were cultured in Dulbecco's Modified Eagle Medium, supplemented with 2 mM L-glutamine and 10% fetal bovine serum (FBS). Human Umbilical Vein Endothelial Cells (HUVEC; Lonza, Walkensville, MD, USA) were cultured in EGM™-2 media (Lonza) and BulletKit™ supplement (Lonza). HL-60 (ATCC) cells were cultured in Roswell Park Memorial Institute (RPMI) 1640, containing 20% FBS (TCB, Long Beach, CA, USA) and differentiated with complete media, containing 1.5% dimethyl sulfoxide (DMSO) for 5 days. THP-1 (ATCC) cells were cultured in RPMI 1640, containing 10% FBS. All cells were maintained in a 5% CO_2_ incubator at 37°C. Primary neutrophils were isolated from femurs and tibias of mice using 65 and 72% Percoll® (Sigma-Aldrich) two-layer gradients. After centrifugation, recovered cells were incubated with red blood cell lysis buffer (ACK Lysing Buffer, Gibco, Waltham, MA, USA). The purity of isolated primary neutrophils (CD11b^+^GR1^+^) was determined to be >90% (data not shown).

### Evans Blue Leakage Assay

Evans blue (50 mg/kg, Sigma-Aldrich) was diluted in PBS and injected intravenously into mice 30 min before sacrifice. After sacrifice, mice were perfused by right ventricle puncture with PBS, and lungs were photographed. Following drying at 56°C for 48 h, lungs were weighed, and Evans blue dye was extracted in 500 μl of formamide (Sigma-Aldrich). The absorbance of these supernatants was measured by spectrophotometry (Molecular Devices, Sunnyvale, CA, USA) at a wavelength of 620 nm. Evans blue concentrations were calculated as extracted Evans blue concentration (ng) divided by the dry lung tissue weight (mg) and compared to measurements from a standard curve.

### Hematoxylin and Eosin Staining and Immunohistochemistry

Lung tissue specimens were fixed in 10% buffered formalin for 24 h, embedded in paraffin, and sectioned at 4 μm. Tissue sections were stained with hematoxylin and eosin (H&E). For immunohistochemistry (IHC) analyses, 4-μm thick lung serial sections were cut and mounted on charged glass slides (Superfrost Plus; Fisher Scientific, Rochester, NY, USA). The sections were deparaffinized and then treated with 3% hydrogen peroxide in methanol to quench the endogenous peroxidase activity. Samples were then incubated with 1% bovine serum albumin (BSA; Gibco) to block non-specific binding. After blocking, sections were incubated with primary rat anti-neutrophil (NIMP-R14, Thermo Fisher Scientific Inc., Waltham, MA, USA) antibody (1:100) or mouse anti-LPS (Abcam, Cambridge, UK) antibody (1:100) at 4°C overnight. After washing, the slides were incubated with a 1:250 dilution of secondary antibody, either horseradish peroxidase-conjugated goat-anti-rat IgG (Santa Cruz Biotechnology, Dallas, TX, USA) or horseradish peroxidase-conjugated goat-anti-mouse IgG (Dako, Santa Clara, CA, USA), at room temperature for 15 min. Images were observed under light microscopy (Olympus, Shinjuku, Tokyo, Japan).

### Histological Scoring and Myeloperoxidase Activity Assay

Lung injury scores were measured by a blinded investigator using published criteria (Table 1 and Equation 1), which are based on neutrophil infiltration (in the alveolar or the interstitial space), hyaline membranes, proteinaceous debris filling the airspaces, and septal thickening (20). To measure myeloperoxidase (MPO) activity in ALI mice, lungs were isolated and homogenized with 0.1% IGEPAL® CA-630 (Sigma-Aldrich). After centrifugation for 30 min, MPO activity was determined using the Myeloperoxidase Activity Assay Kit (Abcam). Sample absorbance was measured using a microplate reader (Molecular Devices) at 410 nm.

(1) $$\text{The final score}=\frac{\left[(20\times \text{A})\;+\;(14\times \text{B})\;+\;(7\times \text{C})\;+\;(7\times \text{D})\;+\;(2\times \text{E})\right]}{\text{Number of fields}\times 100}$$

**Table 1:** Lung injury scoring criteria from Matute-bello et al. (20).

**Parameter**	**Score per field**		
	**0**	**1**	**2**
A. Neutrophils in the alveolar space	None	1–5	>5
B. Neutrophils in the interstitial space	None	1–5	>5
C. Hyaline membranes	None	1	>1
D. Proteinaceous debris filling the airspaces	None	1	>1
E. Alveolar septal thickening	<2x	2x−4x	>4x

### Reverse Transcription Polymerase Chain Reaction (RT-PCR) and Real-Time PCR

Total RNA was extracted using Total RNA Extraction Solution (Favorgen, Taiwan), according to the manufacturer's instructions. This RNA was used in reverse transcription reactions with oligo-dT primers and M-MLV RT reagents (Promega, Madison, WI, USA), according to the manufacturer's instructions. For RT-PCR, the synthesized cDNA was mixed with 2x PCR Master Mix (Solgent, Daejeon, Republic of Korea) and 10 pmol specific PCR primer pair following the manufacturer's protocol. The primers were synthesized from Macrogen (Seoul, Republic of Korea; see Table 2 for primer sequences). Amplified products were separated on 1% agarose gels, stained with ethidium bromide, and photographed under UV illumination using a GelDoc (Bio-Rad Laboratories, Hercules, CA, USA).

**Table 2:** Primers used for PCR.

	**Sense primer**	**Antisense primer**
MIP-2	AGT GAA CTG CGC TGT CAA TG	CTT TGG TTC TTC CGT TGA GG
S100A8	ATG CCG TCT GAA CTG GAG AA	TGC TAC TCC TTG TGG CTG TC
S100A9	ATG GCC AAC AAA GCA CC TT	TTA CTT CCC ACA GCC TTT GC
GAPDH	CCA TCA CCA TCT TCC AGG AG	ACA GTC TTC TGG GTG GCA GT
TRIF	TGT TGG AAA GCA GTG GCC TAT	GAT GAC GTG GTG TTC TGC AGA
TRAM	AGG CTA CAC AGA GAA ACC CC	TGT GAC TTC CTG GCC ATG AT
TIRAP	GAT CGT CAC CAG CTT CCA TT	CCT GAT GCC AGA GGA AGA AG
Myd88	TCG AGT TTG TGC AGG AGA TG	AGG CTG AGT GCA AAC TTG GT
IFNβ	AAG AGT TAC ACT GCC TTT GCC ATC	CAC TGT CTG CTG GTG GAG TTC ATC
IL-1β	TGT AAT GAA AGA CGG CAC ACC	TCT TCT TTG GGT ATT GCT TGG
TNF	ATG AGA AGT TCC CAA ATG GC	CTC CAC TTG GTG GTT TGC TA
LPL	GGG CTC TGC CTG AGT TGT AG	GTC AGG CCA GCT GAA GTA GG
GPI-HBP1	AGC AGG GAC AGA GCA CCT CT	AGA CGA GCG TGA TGC AGA AG
Clathrin	GGG CAA ATC AAA GAA GTG GA	GAG CAG TCA ACA TCC AGC AA
Caveolin-1	ACC TCT CTG GAC TGG CAG AA	GGA AAG GTC GAG CTT CAC AG
IL-6	GAT GCT ACC AAA CTG GAT AT	GGT CCT TAG CCA CTC CTT CTG TG
CXCL1	AGA CTG CTC TGA TGG CAC CT	CTG CAC TTC TTT TCG CAC AA
CXCL3	CAA CGG TGT CTG GAT GTG TC	AGC CAA GGA ATA CTG CCT TA
CXCL5	GTA TCC TGG GTT TCC GGA CT	GAT CTC CAT CGC TTT CTT CG
CXCL12	GAG CCA ACG TCA AGC ATC TG	CGG GTC AAT GCA CAC TTG TC
CCL2	CCC AAT GAG TAG GCT GGA GA	AAA ATG GAT CCA CAC CTT GC
CCL3	CCA AGT CTT CTC AGC GCC AT	TCC GGC TGT AGG AGA AGC AG
CCL4	TCT TGC TCG TGG CTG CCT	GGG AGG GTC AGA GCC CA
CCL5	ATA TGG CTC GGA CAC CAC TC	AGC AAG CAA TGA CAG GGA AG
CCL7	GTG TCC CTG GGA AGC TGT TA	TCC TTA GGC GTG ACC ATT TC
CCR1	AAG GCC CAG AAA CAA AGT CT	TCT GTA GTT GTG GGG TAG GC
CCR2	CCT GCA AAG ACC AGA AGA GG	GTG AGC AGG AAG AGC AGG TC
CCR3	AAG GAC TTA GCA AAA TTC AC CA	ACA CCA GGG AGT ACA GTG GA
CCR5	CGT TCC CCC TAC AAG AGA CT	ACC CAC AAA ACC AAA GAT GA
CXCR1	TCA GTG GTT CCT GCT GC TG	GCA GAC GAG GAT AGT GAG CA
CXCR2	GAT GTC TAC CTG CTG AAC CT	ACC AGG TTG TAG GGC AGC CA
CXCR4	GGG GAC ATC AGT CA GG	GTG GAA GAA GGC GAG GG

A SYBR Green kit (Applied Biosystems, Foster City, CA, USA) was used for real-time PCR (qPCR) analysis of cDNA according to the manufacturer's instructions. Thermal cycling conditions were as follows: initial denaturation at 95°C for 15 min, followed by 40 cycles of 95°C for 30 s, 60°C for 30 s, and 72°C for 30 s. A melting step was performed by raising the temperature from 72 to 95°C after the last cycle. Thermal cycling was conducted on a ViiA 7 Real-Time PCR System machine (Applied Biosystems). The target gene expression levels are shown as a ratio in comparison with GAPDH expression in the same sample by calculation of cycle threshold (Ct) value. The relative expression levels of target genes were calculated by the 2^−ΔΔCT^ relative quantification method.

### Enzyme-Linked Immunosorbent Assay (ELISA)

Concentrations of MIP-2, IFN-β, IL-1β, and TNF were measured using ELISA kits for MIP-2 (R&D Systems, Minneapolis, MN, USA), mouse IFN-β (R&D Systems), mouse IL-1β (BD Biosciences, Franklin Lakes, NJ, USA), and mouse TNF (BD Biosciences), according to the manufacturers' instructions. Cytokine levels were estimated by interpolation from a standard curve generated using an ELISA reader (Molecular Devices) at 450 nm.

### Immunofluorescence Staining and Flow Cytometric Analysis

To detect TLR4/MD2 on membrane surfaces, cells were fixed with 2% paraformaldehyde (Sigma-Aldrich) and blocked with PBS, containing 1% BSA (Gibco). Cells were then incubated with rabbit anti-TLR4/MD2 antibody (Thermo Fisher Scientific Inc.) and Alexa488-conjugated anti-rabbit IgG (Invitrogen, Carlsbad, CA, USA) without permeabilization. For detection of p47phox, Rac-1, lysosomes, ROS, and intracellular LPS, cells were fixed and permeabilized. Samples were then stained with rabbit anti-p47phox (Thermo Fisher Scientific Inc.), mouse anti-Rac-1 (Merck Millipore, Billerica, MA, USA), FITC-conjugated CM-H2DCFDA (Invitrogen) for ROS, the Texas red-conjugated LYSO-ID® Red Detection Kit (Enzo Life Sciences, Inc.), or mouse anti-LPS (Abcam) antibodies, respectively. Secondary antibody staining was then performed with Alexa488-conjugated anti-rabbit IgG (Invitrogen) or Alexa594-conjugated anti-mouse IgG (Thermo Fisher Scientific Inc.). For confocal microscopy analysis, cells were washed with PBS and mounted in 4',6-diamidino-2-phenylindole (DAPI)-containing fluorescence microscopy mounting medium (Invitrogen). Samples were analyzed with a laser scanning confocal microscope (Carl Zeiss, Oberkochen, Germany). For flow cytometric analysis, cells were washed with PBS and analyzed with a FACSVerse Flow Cytometer (BD Biosciences), and the data were processed with FlowJo software (Tree Star, OR, USA).

### siRNA Transfection

Specific siRNA targeting TRIF (sc-154266), TRAM (sc-44748), TIRAP (sc-44740), Myd88 (sc-35987), lipoprotein lipase (LPL) (sc-44900), glycosylphosphatidylinositol high density binding protein 1 (GPI-HBP1) (sc-145686), clathrin (sc-35067), and caveolin-1 (sc-29241) were purchased from Santa Cruz Biotechnology. Scramble siRNA (sc-37007) used as control. Cells were transfected with the indicated siRNA duplex targeting constructs (50 nM) and HiPerFect Transfection Reagent (QIAGEN, Hilden, Germany). After incubation for 24 h, downregulation of target gene expression was evaluated by RT-PCR.

### Immunoprecipitation

For immunoprecipitations, cells were extracted in lysis buffer containing 25 mM Tris-HCl, 150 mM NaCl, 1 mM EDTA, 1% NP-40, and 5% glycerol with protease inhibitor (Roche, Indianapolis, IN, USA). Anti-TLR4/MD2 antibody (Thermo Fisher Scientific Inc.) (2 μg) was added to 200 μl of each cellular extract, and these were incubated at 4°C for 6 h on a rotator. Pre-washed protein G beads (Bio-Rad) (100 μl) were then added to each sample, followed by incubation at 4°C for 4 h. Samples were washed three times in PBS-T [PBS, containing 0.05% Tween-20 (Merck Millipore, Billerica, MA, USA)], solubilized in Laemmli buffer at 70°C for 10 min, boiled for 10 min, and verified by western blot.

### Western Blot Analysis

Cells were ruptured on ice with 1x RIPA lysis buffer (Cell Signaling Technology, Danvers, MA, USA), containing protease inhibitor (Roche) and phosphatase inhibitor (Thermo Fisher Scientific Inc.). Cell lysates were clarified by centrifugation, and samples were analyzed by sodium dodecyl sulfate-polyacrylamide gel electrophoresis (SDS-PAGE). Proteins in gels were transferred onto polyvinylidine (PVDF) membranes (Bio-Rad), and these were blocked with 5% BSA (Gibco) in PBS-T. Membranes were then incubated with antibodies against TRIF (Abcam), Myd88 (Cell Signaling Technology), TLR4 (Invitrogen), phospho-TRAM (MyBioSource, San Diego, CA, USA), TRAM (R&D Systems), phospho-TIRAP (Y86; Abcam), TIRAP (Abcam), phospho-IRF3 (Ser396; Cell Signaling Technology), IRF3 (Cell Signaling Technology), phospho-p65 (Ser536; Cell Signaling Technology), and p65 (Enzo Life Sciences, Inc.) overnight at 4°C. After washing with PBS-T, membranes were stained with peroxidase-conjugated goat anti-rabbit IgG (Santa Cruz Biotechnology) or peroxidase-conjugated goat anti-mouse IgG (Santa Cruz Biotechnology). Target proteins were then detected using the Immobilon Western Chemiluminescent HRP Substrate (Merck Millipore).

### *In vitro* Permeability Assay and Cell Migration Assay

To measure the permeability of endothelial cells *in vitro*, HUVEC cells (1 × 10^4^) were seeded onto 5-μm pore Transwell inserts (Corning Inc., Corning, NY, USA) in complete media. After 16 h, supernatants were removed, and 5 × 10^4^ differentiated HL-60 (dHL-60) cells were loaded onto the HUVEC cells into the top Transwell chamber. THP-1 cells were pre-incubated with PLAG (100 μg/ml) for 1 h and then stimulated with LPS (100 ng/ml). After 16 h, these were centrifuged, and the supernatant was transferred into the bottom Transwell chambers. The combined Transwells were incubated at 37°C for 6 h, after which migrated dHL-60 cells were counted using a hemocytometer and trypan blue staining. Transwell assays were also used to verify albumin efflux into the alveolar compartment observed in the *in vivo* animal model. Briefly, in place of albumin, streptavidin-HRP was laid on the upper chamber with HL-60 cells for 5 min, and then the medium (100 μl) containing transmigrated HRP in the lower chamber was collected and assayed for activity using 100 μl of 3,3',5,5'-tetramethylbenzidine (TMB) substrate (Surmodics, Eden Prairie, MN, USA). Color development was measured by microplate reader (Molecular Devices) at 450 nm.

To measure migration of primary neutrophils, Raw264.7 cells were pre-incubated with PLAG (100 μg/ml) for 1 h and stimulated with LPS (100 ng/ml) for 16 h. Cells were centrifuged, and the supernatant was transferred to the bottom chamber of a Transwell plate. Isolated mouse neutrophils were suspended in RPMI 1640 without FBS, and loaded onto 3 μm-pore Transwell filters (Corning) positioned on top of the migration chamber. Combined Transwells were incubated at 37°C for 2 h, and migrated neutrophils were counted using a hemocytometer with trypan blue staining.

### Statistical Analysis

Results are presented as the mean ± standard error of the mean (s.e.m.). The level of significance, assumed at the 95% confidence limit or greater (*p* < 0.05), was calculated with one-way analysis of variance (ANOVA), followed by Duncan's *post hoc* test, using SPSS software; ^*^ indicates *p* < 0.05.

## Results

### PLAG Resolves LPS-Induced ALI Through Regulation of Excessive Neutrophil Infiltration

**Figure 1:**
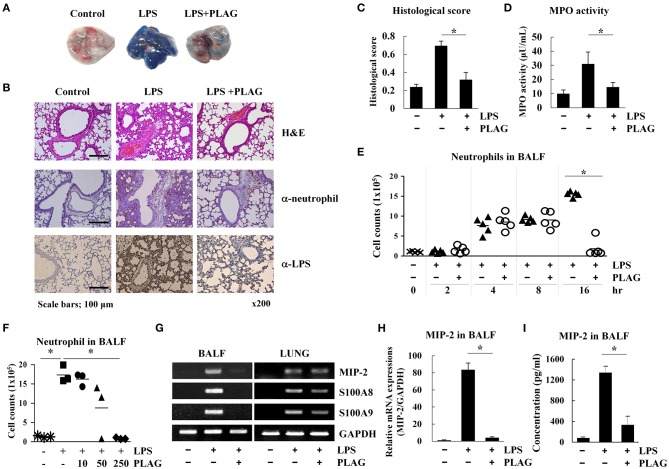
PLAG suppresses lung inflammation in a mouse model of acute lung injury (ALI) through regulation of neutrophil infiltration. Mice were divided into four separate groups (*n* = 5 per group): control, LPS-treated, and PLAG/LPS co-treated. LPS (25 mg/kg) was intranasally injected, and PLAG (250 mg/kg) was administered orally. Evans blue dye (50 mg/kg) was injected intravenously 30 min before sacrifice following LPS or PLAG/LPS treatment for 16 h, and lungs were harvested from all animals. Representative lungs demonstrating Evans blue accumulation are shown **(A)**. Histological examination of lung tissues was performed 16 h after LPS administration. Lung sections were stained with HandE or with neutrophil and LPS-specific antibodies **(B)**. Lung injury scoring was calculated as described in Table 1 and Equation 1 **(C)** (20). Lungs from LPS and PLAG/LPS-treated animals, as well as controls, were examined for MPO activity **(D)**. Following LPS or PLAG/LPS stimulation for 2, 4, 8, and 16 h, mice were sacrificed, and the number of neutrophils in bronchoalveolar lavage fluid (BALF) was counted using complete blood count (CBC) analysis **(E)**. The bar represents the mean. Mice were divided into five separate groups (*n* = 3 per group): control, LPS-treated, PLAG (10, 50, 250 mg/kg)/LPS co-treated. LPS (25 mg/kg) was intranasally injected and PLAG was administered orally. Following LPS for 16 h, mice were sacrificed, and the number of neutrophils in bronchoalveolar lavage fluid were counted using complete blood count (CBC) analysis **(F)**.Total RNA was extracted from BALF cells and homogenized lungs and analyzed by reverse transcription (RT)-PCR **(G)** and real-time PCR (qPCR) **(H)**. GAPDH was used as a control. Levels of secreted MIP-2 in BALF were measured using an ELISA assay **(I)**. All *in vivo* data were obtained from at least three independent experiments with five mice for each group **(A–G)**. Data shown represent one experiment performed in triplicate. ^*^indicates *p* < 0.05.

LPS is able to recruit immune cells into the lung alveolar compartment and promotes the secretion of inflammatory mediators. Thus, it is commonly used to induce development of ALI in a mouse model (21). Evans blue dye extravasation into the tissue can further used as an index of increased vascular permeability and neutrophil transmigration in ALI and control mice (22). Here, we used this Evans blue leakage assay to investigate the effects of PLAG (administered orally) on vascular leakage in mice that were intranasally administered with LPS. We found that in mice treated with LPS alone for 16 h, lung tissues showed excessive leakage of albumin from blood vessels to the alveolar space, as demonstrated by increased Evans blue staining (Figure 1A). Lungs from mice co-treated with PLAG/LPS, however, showed decreased Evans blue-stained albumin. These findings were confirmed by a quantitative analysis of Evans blue-labeled albumin extract from the lungs (Supplementary Figure 1a), which shows a decreased level of Evans blue dye in lungs from mice treated with PLAG/LPS, as compared to LPS alone.

A high level of Evans blue staining is correlated with the vast extravasation of neutrophils into the alveolar space. We therefore, examined the effect of PLAG on leukocyte cell infiltration into the lung alveolar compartment using H&E staining. These data revealed that intranasal LPS administration induces extensive inflammatory cell infiltration into the lung tissue compared to control animals. However, PLAG/LPS co-treated mice exhibit a considerably reduced inflammatory cell infiltration into the alveolar space and display normal alveolar morphology (Figure 1B). Histological scores of control, LPS, and PLAG/LPS-treated mice show the same effect (Figure 1C, Table 1, and Equation 1).

An increase in MPO activity reflects neutrophil accumulation in the lungs. Here, we found that MPO activity of isolated lung tissue is substantially increased in LPS-treated mice but is significantly decreased in the PLAG/LPS co-treated mice, as compared to those treated with LPS alone (Figure 1D). These data suggest that PLAG plays a protective role in ALI by blocking excessive neutrophil influx into the lung tissue. To further test this hypothesis, neutrophils in BALF were counted 2, 4, 8, and 16 h after LPS intranasal administration (Figure 1E). We found that LPS challenge significantly increases neutrophil infiltration time-dependently into BALF compared to the control. However, PLAG/LPS co-treated animals more rapidly return to homeostasis, showing baseline numbers of neutrophils in BALF by 16 h post-treatment. The amount of neutrophils transmigrated to the BALF at 16 h after LPS intranasal injection decreased in a dose-dependent manner in pretreated PLAG (10, 50, 250 mg/kg) (Figure 1F). PLAG treatment alone has no effect on neutrophil migration, and PLAG/LPS co-treatment does not alter neutrophil release from bone marrow or apoptosis (Supplementary Figures 1b–e). Thus, these data indicate that PLAG can specifically modulate excessive neutrophil infiltration into the lung.

To more precisely determine the role of PLAG in controlling excessive neutrophil infiltration into lung tissue in our ALI model, we measured the expression of several inflammation-related molecules in BALF cells and lung-homogenized tissue after treatment with PLAG and/or LPS for 16 h. We found that mRNA expression levels of MIP-2 [CXC Chemokine Ligand 2 (CXCL2)], a main factor involved in neutrophil migration, as well as S100A8 and S100A9, are increased in BALF cells from mice treated with LPS for 16 h compared to those from control animals (Figures 1G–I and Supplementary Figure 2c). This increased gene expression, however, is significantly attenuated in mice co-treated with PLAG/LPS for 16 h. Additionally several chemokines such as CXCL5, C-C motif chemokine ligand 2 (CCL2), or CCL5 exhibit a significant decrease of mRNA levels following treatment of PLAG (Supplementary Figures 2b,c). However, the reduction of CXCL5 and CCL2 mRNA expression after LPS/PLAG co-treatment was a small difference compared with the difference of the decrease expression levels of MIP-2 gene in BALF cells. The mRNA expression levels of CCL5, a key chemokine involved monocytes/macrophages migration is increased in BALF cells by LPS treatment and significantly decreased in mice co-treated with PLAG/LPS for 16 h. Other chemokines and their receptors exhibit either a moderate decrease or no change following treatment with PLAG (Supplementary Figures 2a,b). Further, we found that secreted levels of MIP-2 are also significantly increased in BALF following LPS administration, and markedly decreased by co-treatment with PLAG (Figure 1I). We have confirmed that inhibitors of CXC chemokine receptor 2 (CXCR2), a MIP-2 binding receptor, also completely reduced neutrophil infiltration to the lung of mice with LPS treatment (data not shown). The major inflammatory cytokine, IL-6 was also decreased in BALF and serum with PLAG administration (Supplementary Figure 2d). It is reported that LPS-induced TLR4 stimulation increases IL-6 expression (23). Thus, these results suggest that PLAG exerts an anti-inflammatory effect by blocking excessive neutrophil infiltration, at least in part, through the modulation of MIP-2 expression.

### PLAG Induces More Rapid Endocytosis and Recovery of TLR4 Than LPS Alone and Promotes Clearance of Engulfed LPS

**Figure 2:**
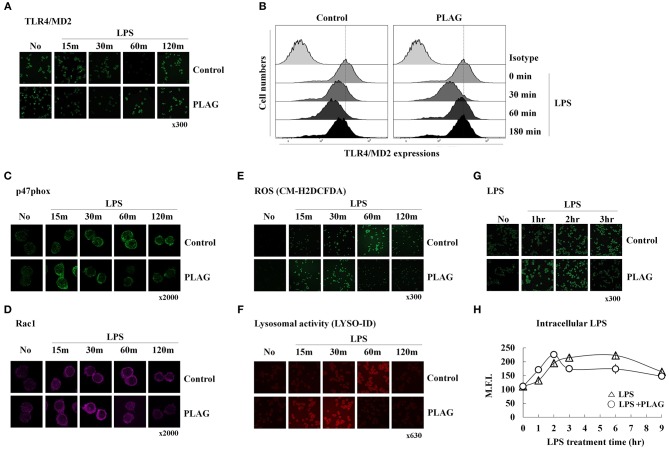
PLAG accelerates LPS-induced TLR4 endocytosis and rapid LPS clearance. Raw264.7 cells were treated with 100 μg/ml of PLAG or DMSO (as solvent control) for 1 h and treated with 100 ng/ml LPS for 15, 30, 60, 120, and 180 min. Cells were then fixed and stained using rat anti-TLR4/MD2 antibody with Alexa488-conjugated anti-rat IgG secondary antibody. These were analyzed by confocal microscopy **(A)** and flow cytometry **(B)**. Raw264.7 cells stimulated under the same conditions were fixed, permeabilized, and stained with rabbit anti-p47phox **(C)** and mouse anti-Rac1 **(D)**, CM-H2DCFDA **(E)**, or the LYSO-ID Lysosomal Detection Kit **(F)**. Confocal microscopy was performed; all data shown represent one experiment performed in triplicate. Raw264.7 cells were treated with 100 μg/ml of PLAG or DMSO (as solvent control) for 1 h and stimulated with 100 ng/ml LPS for 1, 2, 3, 6, and 9 h **(G,H)**. Cells were then fixed, permeabilized, and stained with mouse anti-LPS antibody and Alexa488 conjugated anti-mouse IgG secondary antibody. These were analyzed by confocal microscopy and flow cytometry. Data represent one experiment performed in triplicate.

We next investigated the role of PLAG in ALI protection using an *in vitro* cell culture system. In RAW264.7 cells, a mouse macrophage cell line, LPS stimulates its cognate receptor TLR4 and subsequently induces LPS engulfment with aid of this receptor. Here, we evaluated the effect of PLAG on internalization of the LPS/TLR4 complex by analysis of surface spanning TLR4 using anti-TLR4/MD2 antibodies. PLAG-treated Raw264.7 cells show a more rapid endocytosis of the LPS/TLR4 complex and earlier recovery of TLR4 on surface membranes than those treated with LPS alone (Figures 2A,B). Specifically, initiation of TLR4 internalization can be clearly observed 30 min after treatment with LPS alone, whereas internalization is apparent after 15 min in cells co-treated with PLAG/LPS. Similarly, return of the TLR4 receptor to the cell surface membrane occurs 120 min after treatment with LPS and 60 min after co-treatment with PLAG/LPS. These data show that PLAG accelerates internalization of TLR4 receptor and promotes its return to the surface membrane.

We next investigated whether accelerated LPS/TLR4 endocytosis could lead to a more rapid clearance of intracellular LPS. It is well-known that, in macrophages, internalized LPS spontaneously stimulates generation of ROS, which function to eliminate the source of intracellular LPS. This also activates signaling pathways leading to production of numerous chemokines (mainly MIP-2) that recruit circulating neutrophils to the infection site. ROS generation is closely regulated by the NADPH oxidase system (24). Here, we found that in LPS-stimulated Raw264.7 cells, recruitment of p47phox, and Rac1 from the cytosol to the membrane can be observed at 30 min post-treatment and is sustained until 120 min post-treatment. In PLAG/LPS co-treated cells, the recruitment and return to homeostasis of both p47phox and Rac1 are observed at 15 and 60 min, respectively (Figures 2C,D). Similarly, ROS production can be detected 15 min earlier in PLAG/LPS-treated cells compared to those treated with LPS alone, and return to homeostatic levels of intracellular ROS occurs at 60 and 120 min post-treatment, respectively, for the PLAG/LPS- and LPS-treated groups (Figure 2E). These results were further confirmed using a lysosomal activity detection kit (Figure 2F). Thus, our data reveal that PLAG accelerates endocytosis of LPS/TLR4 and promotes a more rapid recruitment of p47phox and Rac1 enzymes and ROS production. In addition, return to homeostasis occurs more rapidly PLAG co-treated cells. To determine the effect on LPS clearance, we measured levels of intracellular LPS and found both a more rapid uptake and a faster removal of LPS in PLAG-treated cells (Figures 2G,H). Our data therefore indicate that PLAG facilitates a more rapid LPS-induced TLR4 endocytosis and accelerates LPS-induced ROS production and return to homeostasis via earlier clearance of LPS from invading pathogen.

### PLAG Works as a Vesicle and Advanced TLR4 Endocytosis by PLAG Was Dependent on LPL and GPI-HBP1

**Figure 3:**
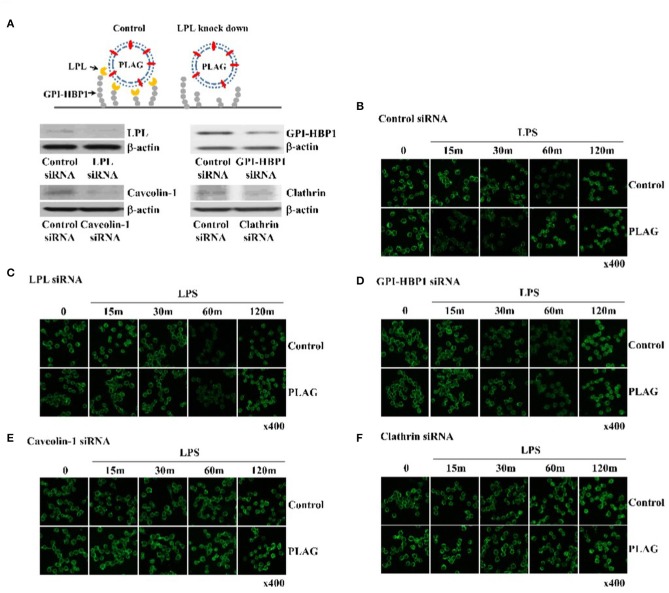
PLAG has an effect on accelerated TLR4 endocytosis via LPL and GPI-HBP1 dependent pathway. Raw264.7 cells were transfected with LPL, GPI-HBP1, caveolin-1, or clathrin siRNA, respectively. After 24 h, LPL, GPI-HBP1, caveolin-1, or clathrin levels were assessed by western blot **(A)** and cells were incubated with 100 ug/ml of PLAG or DMSO (as solvent control) for 1 h. LPS was treated with 100 ng/ml for 15, 30, 60, 120 min, cells were fixed, permeabilized, and stained using rat anti-TLR4/MD2 antibody and Alexa488 conjugated anti-rat IgG as secondary antibody. Cells were analyzed confocal analysis **(B–F)**. β-actin was used as a control. All data shown represent one experiment performed in triplicate.

PLAG is acetylated DAG with palmitic and linoleic acid and spontaneously forms a vesicle under emulsification (Figure 3A). Vesicle is recognized by LPL secreted by peripheral tissue resident cells. LPL bound vesicle is trapped by GPI-HBP1. GPI-HBP1 is expressed in the most cell waiting for uptake of lipid molecules as an energy source. To verify PLAG has role as a vesicle in the rapid endocytosis and recovery of TLR4, Knockdown of LPL and GPI-HBP-1 in THP-1 cells was constructed by siRNA transfection. Gene silencing of LPL, GPI-HBP1, caveolin-1, and clathrin was confirmed by western blot analysis (Figure 3A). In the scramble RNA transfected cells as control, PLAG/LPS treatment enables to rapid endocytosis at 15 min and recovery of TLR4 at 60 min when compared to only LPS treatment (Figure 3B). In the LPL or GPI-HBP1 gene silenced cells, added PLAG effect on rapid endocytosis and recovery of TLR4 was not observed (Figures 3C,D). In the caveolin-1 or clathrin silenced cells, endocytosis of TLR4 was not found (Figures 3E,F). These results suggest that PLAG is working as a form of micelles and has biological effects on the peripheral tissue containing cells which express LPL, GPI-HBP-1.

### PLAG Affects TRIF-Dependent Endosomal Signaling Rather Than the Myd88 Pathway After LPS Stimulation

**Figure 4:**
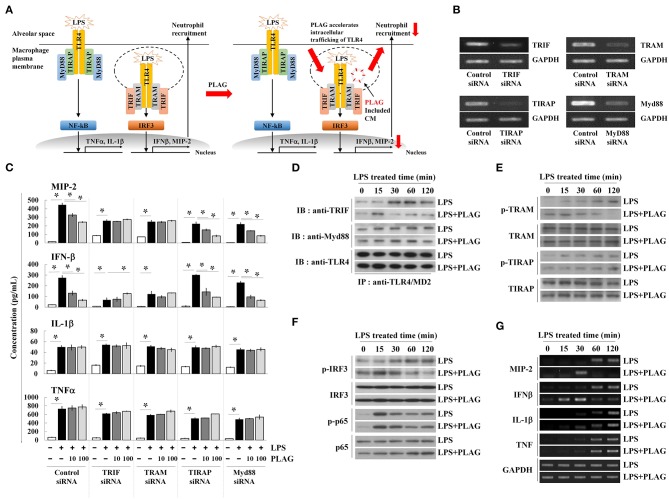
PLAG accelerates the LPS-induced TRIF-dependent endocytosis pathway. Schematic diagram of TLR4-mediated signaling in response to LPS and PLAG/LPS in macrophages. TLR4 endocytosis by LPS treatment occurs and MIP-2 is produced by internalization signaling of TLR4, and neutrophils enter the alveoli. In the macrophages treated with PLAG, TLR4 intercellular trafficking by LPS is promoted and the internalization signaling of TLR4 is terminated rapidly, resulting in decreased expression of MIP-2, and reduction of neutrophil infiltration **(A)**. Raw264.7 cells were transfected with siRNA targeting TRIF, TRAM, TIRAP, and Myd88, as well as the scrambled control. After 24 h, target mRNA levels were assessed by RT-PCR **(B)**. Transfected cells were then incubated with 10 or 100 μg/ml of PLAG or DMSO (as solvent control) for 1 h and then stimulated with 100 ng/ml LPS for 16 h. Culture supernatants were assayed by ELISA to measure the secreted levels of MIP-2, IFN-β, IL-1β, and TNF **(C)**. Raw264.7 cells were incubated with 100 μg/ml of PLAG or DMSO (as solvent control) for 1 h and then stimulated with 100 ng/ml LPS for 15, 30, 60, and 120 min. Following immunoprecipitation with TLR4/MD2 antibodies, cell lysates were separated by SDS-PAGE and analyzed by immunoblot analysis with antibodies to TRIF, Myd88, and TLR4 **(D)**. The whole lysates were analyzed by immunoblot analysis to assess phosphorylation of TRAM or TIRAP **(E)** and IRF3 or p65 **(F)**. Total mRNA was extracted from whole cells, and expression of MIP-2, IFN-β, IL-1β, and TNF was assessed by RT-PCR **(G)**. GAPDH used as loading control. All Data represent one experiment performed in triplicate; ^*^indicates *p* < 0.05.

LPS binding to the TLR4/MD2 complex activates two distinct signaling pathways; the Myd88-dependent network is associated with membrane-localized TLR4 receptors, whereas the TRIF-dependent pathway is associated with endosomal signaling (Figure 4A) (25). Above, we observed that treatment with PLAG attenuates MIP-2 expression in lung tissue and BALF from LPS-treated mice, which is mainly dependent on the TRIF adaptor protein (Figures 1G–I). Therefore, to determine whether PLAG modulates the TRIF- and/or the MyD88 dependent pathways, Raw264.7 cells were transfected with specific siRNAs targeting Myd88 and TIRAP, to block Myd88-dependent signaling, and siRNAs for TRIF and TRAM, to inhibit TRIF-dependent signaling; target mRNAs were all effectively downregulated in siRNA-transfected cells (Figure 4B). Notably, in TRIF- and TRAM-silenced cells, the ability of PLAG to modulate production of MIP-2 and IFN-β is completely abolished (Figure 4C). However, in cells silenced for TIRAP or Myd88, the effects of PLAG effect could still be observed in a dose-dependent manner. This suggests that the ability of PLAG to modulate MIP-2 and IFN-β expression is mainly dependent on TRIF and the endosomal-associated signaling pathway. In contrast, LPS-induced expression of IL-1β and TNF, which is mediated by membrane-localized TLR4 receptor, is not modulated by PLAG (Figure 4C).

Modulation of TRIF signaling by PLAG in LPS-treated cells was further investigated by assessing the modification of TLR4-associated adaptor molecules. Assembled TLR4 adaptor proteins were precipitated using anti-TLR4/MD2 antibodies in LPS-stimulated cells with or without PLAG. Association of TRIF with TLR4/MD2 was detected at 30 min post-treatment and maintained up to 120 min in LPS-treated cells (Figure 4D). In PLAG/LPS co-stimulated cells, TRIF and TLR4 assembly initiates at 15 min post-treatment and complex disassembly was detected 60 min after LPS treatment. Conversely, association between Myd88 and TLR4 are unchanged in PLAG/LPS- vs. LPS-stimulated cells. These data demonstrate that PLAG accelerates the association and disassociation of TRIF and TLR4.

TRAM activation via phosphorylation recruits the TRIF adaptor molecule to the TLR4 endosomal-signaling pathway (26). Using anti-phospho-TRAM antibodies and western blot analysis, we detected TRAM phosphorylation at 30 min post-treatment, and this is sustained for up to 120 min in LPS-treated cells (Figure 4E). In PLAG/LPS-treated cells, phosphorylation of TRAM was found at 15 min post-treatment and terminates by 60 min. Notably, this earlier activation and faster resolution of TRAM phosphorylation in response to PLAG/LPS stimulation was found to be specific for this molecule, as the phosphorylation of TIRAP is unchanged by addition of PLAG in LPS-stimulated cells (Figure 4E).

IRF3 and NF-κB are the main transcription factors activated in LPS-stimulated cells; these mediate activity of the endosomal TLR4 receptor-dependent signaling pathway and the membrane-localized TLR4 receptor-dependent signaling pathway, respectively. Phosphorylation of IRF3 in response to LPS stimulation can be detected at 30 min post-treatment and is maintained up to 120 min (Figure 4F). Co-treatment with PLAG, however, accelerates IRF3 phosphorylation, resulting in a peak at 15 min post-treatment, followed by a return to baseline 60 min after co-stimulation. Consistent with these data, MIP-2 and IFN-β mRNA transcripts, which are largely dependent on the TRIF-dependent TLR4 signaling pathway, were detected at 60 min post-stimulation and are sustained for 120 min in LPS-treated cells (Figure 4G). PLAG co-treatment accelerates the appearance of MIP-2 and IFN-β transcripts, and their levels return to control levels by 60 min. Consistent with our siRNA data, we find that PLAG preferentially modulates expression of MIP-2 and IFN-β, as the levels of IL-1β and TNF mRNA are unaffected by PLAG. Collectively, these data suggest that rather than acting as inhibitor, PLAG accelerates the response to LPS, specifically TLR4-mediated endosomal signaling, and returns cells to homeostasis in a shorter period of time.

### PLAG Functions via Micelle Formation in an Endocytosis-Dependent Manner

**Figure 5:**
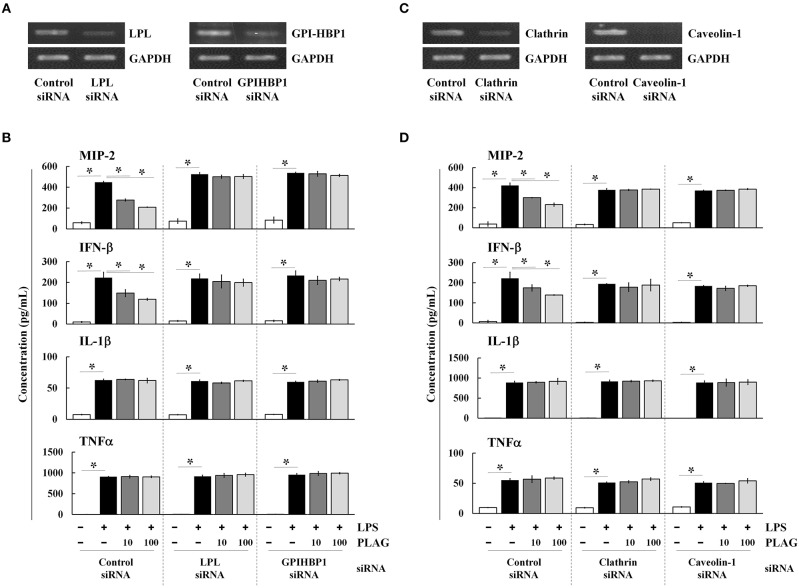
PLAG functions via micelle formation. Raw264.7 cells were transfected with siRNA targeting LPL or GPI-HBP1, and after 24 h, expression of target genes was assessed by RT-PCR **(A)**. Transfected cells were incubated with 10 or 100 μg/ml of PLAG or DMSO (as solvent control) for 1 h and then treated with 100 ng/ml LPS for 16 h. Culture supernatants were assayed by ELISA to measure secreted levels of MIP-2, IFN-β, IL-1β, and TNF **(B)**. Raw264.7 cells were transfected with siRNA targeting clathrin or caveolin-1, and after 24 h, expression of target genes was assessed by RT-PCR **(C)**. Transfected cells were then treated with 10 or 100 μg/ml of PLAG or DMSO (as solvent control) for 1 h and stimulated with 100 ng/ml LPS for 16 h. Culture supernatants were analyzed by ELISA assay to measure secreted levels of MIP-2, IFN-β, IL-1β, and TNF **(D)**. Data represent one experiment performed in triplicate. ^*^indicates *p* < 0.05.

PLAG is an acetylated DAG molecule that is utilized as a structural lipid during micelle formation. Similar to phosphatidylcholine, emulsified PLAG spontaneously forms micelles *in vitro* (data not shown). One type of micelle, known as a chylomicron, is generated in enterocytes and delivered to peripheral tissues where fatty acids in the chylomicron are distributed to cells with the aid of LPL and GPI-HBP1. Dietary PLAG is also absorbed by enterocytes and delivered as a component of chylomicrons into peripheral tissue (data not shown). Because LPS-induced MIP-2 is modulated by PLAG, we hypothesized that modulation of MIP-2 expression in macrophages by PLAG might be dependent on micelle formation. To test this, Raw264.7 cells were transfected with siRNA targeting LPL or GPI-HBP1, and the downregulation of their mRNA levels was confirmed in transfected cells (Figure 5A). We then found that the decreased secretion of MIP-2 and IFN-β observed PLAG/LPS-treated cells as compared to cells treated with LPS alone was not observed in cells silenced for LPL or GPI-HBP1 (Figure 5B); IL-1β and TNF are unaffected. These data suggest that PLAG may function in micelles, which are recognized by LPL and communicate with cells via GPI-HBP1.

We next transfected Raw264.7 cells with siRNAs targeting clathrin and caveolin-1, which are involved in TLR4-mediated endocytosis, and confirmed their downregulation in siRNA-transfected cells by RT-PCR (Figure 5C). As noted above, PLAG co-treatment differentially decreases secretion of MIP-2 and IFN-β in LPS-stimulated cells, but has no effect on IL1-β and TNF (Figure 5D). In clathrin and caveolin-1 silenced cells, however, secretion of MIP-2 and IFN-β is unaffected by PLAG treatment. These data confirm that the biological role of PLAG in the modulation of MIP-2 chemokine expression is dependent on micelle formation and internalization of the TLR4 receptor.

### PLAG Blocks Neutrophil-Mediated Endothelial Permeability *in vitro*

**Figure 6:**
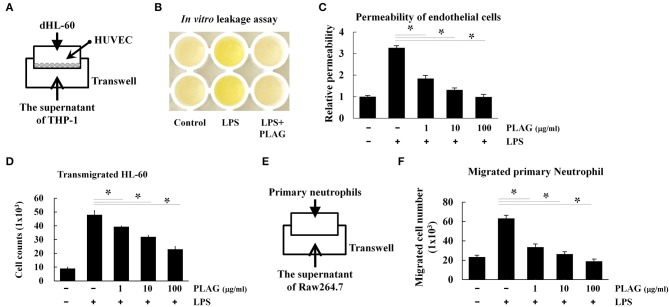
PLAG regulates the excessive vascular leakage of endothelial permeability and neutrophil migration. HUVEC cells (1 × 10^4^) were seeded onto 5-μm pore Transwell inserts, and after 16 h, the supernatant was removed and replaced with media containing differentiated HL-60 cells **(A)**. The lower Transwell chamber was loaded with the supernatant of THP-1 cells that had been pre-incubated with PLAG (100 μg/ml) for 1 h and then stimulated with LPS (100 ng/ml) for 16 h. The combined Transwells were incubated at 37°C for 6 h, and then *in vivo* leakage and endothelial cell permeability were analyzed by the Transwell permeability assay, as described in the Materials and Methods **(B,C)**. Migrated dHL-60 cells were also counted **(D)**. Primary neutrophils were isolated from bone marrow, and their transmigration ability was assessed using supernatant from Raw264.7 cells that were pre-incubated with PLAG (100 μg/ml) for 1 h and then with LPS (100 ng/ml) for 16 h **(E)**. Migrated primary neutrophils were counted **(F)**. All Data represent one experiment performed in triplicate; ^*^indicates *p* < 0.05.

The prominent phenotype of ALI is the massive infiltration of neutrophils into the bronchoalveolar tissues, which results in damage to the endothelial cells and/or loss of the endothelial barrier (27). We therefore investigated whether PLAG protects the integrity of the endothelial barrier using a Transwell assay. For neutrophil cells, we used differentiated HL-60 cells, which were cultured in the upper Transwell chambers. HUVEC cell were plated on the Transwell inserts to act as endothelial barrier cells, and THP-1 cells were used as chemokine-releasing cells. Culture supernatants from LPS-stimulated THP-1 cells contain secreted chemokines, particularly IL-8, and these were placed in bottom Transwell chamber (Figure 6A). In this assay, HL-60 cells in the upper chamber move toward the lower chamber along chemokine gradients, which are provided by the supernatant from the LPS-stimulated THP-1 cells. If neutrophil transmigration occurs, proteins in the upper chamber move across the endothelial HUVEC layer to lower chamber. Loading of streptavidin-HRP in the upper chamber stains the plasma proteins in cell supernatant, similar to Evans blue dye staining of albumin *in vivo*. The migrated plasma proteins in the lower chamber can then be detected by TMB substrate, and this can be used to assess endothelial permeability. Using this assay, we found that supernatant from PLAG/LPS-treated THP-1 cells promote a markedly decreased level of LPS-induced endothelial permeability and dHL-60 cell migration, with PLAG acting in a dose-dependent manner (Figures 6B–D). These results were confirmed by measuring migration of mouse bone marrow-derived primary neutrophils in the same assay (Figures 6E,F). Inhibitors of TLR4 or NOX were further shown to inhibit primary neutrophil transmigration (Supplementary Figure 3). Thus, our data indicate that PLAG can block excessive neutrophil migration into bronchoalveolar tissues.

### Confirmation of PLAG Therapeutic Specificity by Use of PLAG Metabolites

**Figure 7:**
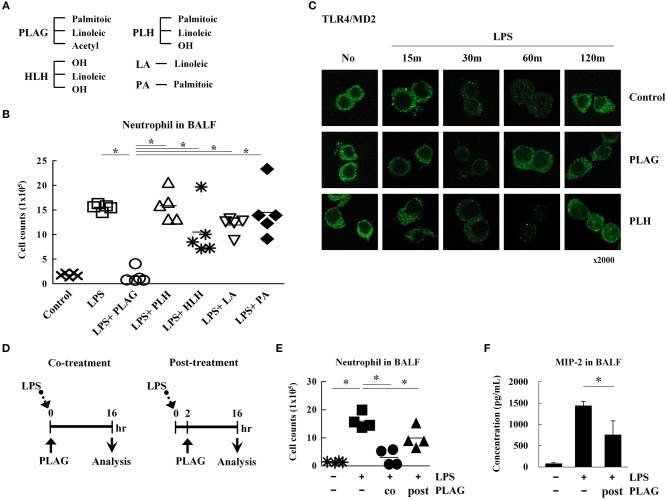
The acetylation of diacylglycerol is critical for attenuating excessive neutrophil infiltration in the ALI model. Chemical structure diagram of PLAG and PLAG metabolites: PLH, HLH, LA, and PA **(A)**. Mice were divided into seven separate groups (*n* = 5 per group): control, LPS-treated, PLAG/LPS co-treated, LPS/PLH co-treated, LPS/HLH co-treated, LPS/LA co-treated, and LPS/PA co-treated. Mice were treated as described in Figure 1A. LPS (25 mg/kg) was administered by an intranasal route, and PLAG (250 mg/kg) or PLAG derivatives (250 mg/kg) were administered orally. After 16 h, BALF cells were counted **(B)**. Raw264.7 cells were incubated with 100 μg/ml PLAG, PLH, or DMSO (as solvent control) for 1 h and then treated with 100 ng/ml LPS for 15, 30, 60, or 120 min. Cells were fixed and stained with rat anti-TLR4/MD2 antibody and Alexa488-conjugated anti-rat IgG secondary antibody **(C)**. Mice were divided into four separate groups: control, LPS-treated, PLAG/LPS co-treated, and PLAG/LPS post-treated (*n* = 4 per group). LPS (25 mg/kg) was administered by an intranasal route, and PLAG (250 mg/kg) was administered orally. In the PLAG/LPS post-treated group, PLAG was administrated after 2 h of LPS treatment **(D)**. Approximately 16 h after LPS administration, mice were sacrificed, and the number of neutrophils in BALF were counted **(E)**. Levels of secreted MIP-2 in BALF were measured using an ELISA assay **(F)**. Data represent four mice samples from each group. *In vivo* experiments were from at least three independent experiments, and the bar represents the mean. ^*^indicates *p* < 0.05.

To determine specificity of PLAG, an acetylated DAG, we assessed the therapeutic efficacy of PLAG metabolites and compared their biological efficacy in our animal model *in vivo*. 1-palmitoyl-2-linoleoyl-3-hydroxyl-rac-glycerol (PLH) is a DAG that consists of two fatty acid chains, palmitic acid and linoleic acid. 1-hydroxyl-2-linoleoyl-3-hydroxyl-glycerol (HLH) is composed of linoleic acid and a glycerol backbone. Linoleic acid (LA) or palmitic acid (PA) was also used (Figure 7A). In our ALI animal model, LPS treatment via intranasal administration induces massive neutrophil extravasation into the alveolar cavity, which is easily detected in the BALF. PLAG co-treated mice show a dramatically reduced number of neutrophils in the BALF, and counts rapidly return to a normal status (Figures 1C, 7B). Conversely, PLH, HLH, LA, and PA have no effect on the number of neutrophils in BALF from LPS-treated mice (Figure 7B). These data indicate that PLAG has a specific role in blocking the excessive and sustained neutrophil infiltration during LPS-induced ALI progression. In LPS-stimulated Raw264.7 cells, TLR4/MD2 internalization is observed at 30 min post-treatment and sustained for up to 120 min. In contrast, PLAG co-treated cells show increased TLR4/MD2 internalization at 15 min, and TLR4 returns to the surface at 60 min. However, co-treatment with PLH has no effect on TLR4/MD2 internalization and return to the surface (Figure 7C). These findings suggest that the acetylation of DAG is a critical factor in blocking excessive neutrophil infiltration in the ALI animal model and.

PLAG exerts a therapeutic effect in the ALI mouse model when co-administered with LPS. Therefore, we next investigated whether PLAG has the same effect when administered post-treatment. To test this, mice were treated with LPS for 2 h, then PLAG was then administrated orally (Figure 7D). We found that the number of infiltrated neutrophils in BALF was diminished by half in the post-treated mice, as compared to those treated with LPS alone (Figure 7E). MIP-2 levels were also significantly decreased in BALF from post-treated group (Figure 7F). Thus, we propose that PLAG may hold potential a therapeutic agent for treatment of inflammatory diseases, such as ALI.

## Discussion

In this study, we tested the therapeutic efficacy of the acetylated DAG, PLAG, for preventing disease pathology in a mouse model of ALI and observed significantly reduced lung inflammation in animals co-treated with PLAG/LPS, as compared to LPS alone (Figure 1). Histological examination showed that severe pulmonary destruction, including bronchial alveolar tissue damage and massive leukocyte infiltration, were observed in the LPS-treated mice. In PLAG/LPS-treated mice, however, LPS-induced ALI was not observed, and the morphology of all lung tissue was similar to that of control mice. In addition, post-treatment with PLAG also reduces the number of infiltrating neutrophils in the alveolar tissue in this ALI mouse model (Figures 7D–F). Further, we found that PLAG, an acetylated DAG that is also found in nature, has a unique biological effect in reducing the amount of infiltrating neutrophils into the alveolar tissue when compared with a natural DAG (PLH), and other PLAG metabolites (Figure 7B). These findings suggest that PLAG might hold potential as a therapeutic agent for prevention of lung inflammation.

PLAG is a synthetic monoacetyldiacylglyceride (17) and a small lipid molecule. In our reports, the concentrations of PLAG in plasma, whole blood, or/and lymph of rat, dog and monkey were obtained measured using the LS-MS/MS method following oral administration of a range of 500 between 4,000 mg/kg of PLAG (unpublished data). Lymphatic exposure of PLAG after oral administration is 10–100-fold increase than blood exposure, and the biggest difference in the PLAG exposure between lymph and blood system was observed in monkeys. Generally, lipid is absorbed through enterocytes, reconstructed with chylomicron, delivered to lymph, and then exposed to blood system, and distributed throughout the body. To sum up the above mentioned results, more than 75% of dosed PLAG was excreted by 24 h and the Cmax 100 mg/kg was 38.8 μg eq./mL in blood and 1,210 μg eq./mL in lymph, respectively. The t1/2 100 mg/kg was 16.8 and 29.7 h, respectively. Based on these results, considering the time when PLAG is sufficiently exposed to the peripheral tissue, it was set at 16 h postdose to see the effects of removing LPS and then resolving systemic inflammation to improve ALI. We have already described in Figure 1E that the basis for selecting the ameliorating time of LPS-induced ALI to reduce neutrophil infiltration to the lung tissue was 16 h after the administration of PLAG. And we have shown that the most effective dose of PLAG in the improvement of ALI was 250 mg/kg. Based on our tests, a simple calculation shows that the concentration of PLAG in the blood of mice administered with 250 mg/kg PLAG is likely to be greater than about 100 μg/mL. Thus, for i*n vitro* assay, the biological effects were verified by treating cells directly at concentrations of 10 to 100 μg/mL PLAG.

Because PLAG is fat, the absorbed PLAG in the body is likely to decompose in the intestine and form metabolites through the recombination process. Therefore, we have checked the metabolite profiling of plasma and lymph fluid. Several peaks in the plasma and the lymph fluid were evaluated quantitatively and qualitatively. In the plasma, the predominant component was suggested to be glucose in the mass spectrometric analysis. In the lymph fluid, the predominant components were suggested to be a mixture of various triglyceride in the mass spectrometric analysis. Unchanged EC-18 and PLH (hydrolyzed form of acetate ester in EC-18) were also detected in the plasma and lymph fluid as minor components. However, we already have checked the effects of the metabolites (e.g., PLH, PA, LA, and HLH) on the neutrophil infiltration to the peripheral lung tissue in LPS-induced ALI model (Figure 7B). The metabolites of PLAG had no effect on the improvement of LPS-induced acute lung inflammation. In addition, the predominant components of the metabolite were suggested to be a mixture of various triglyceride. We have checked the effect of olive oil (composed with 13% saturated fats and >85% unsaturated fats) on the neutrophil infiltration to the inflamed peripheral tissue by the chemical damage (data not shown). Likewise, the administration of olive oil was not affected to improve the tissue inflammation. Taken together, we are convinced that the metabolites have little effect on the improving effects of LPS-induced ALI and that PLAG is the critical effect molecule.

MIP-2 is a known intermediary chemoattractant for neutrophils and plays a key role in inflammation-related diseases, including arthritis, cancers, and pulmonary disease (28–31). LPS stimulation of bronchoalveolar tissue induces secretion of numerous pro-inflammatory molecules, including MIP-2. This chemokine likely contributes to the initiation and extension of inflammatory process and is often abundantly detected at sites of tissue inflammation. Induction of MIP-2 by LPS treatment is mainly controlled by the TLR4-mediated endosome-dependent signaling pathway. Here, we found that PLAG exerts its biological effect by accelerating the endocytosis and exocytosis of TLR4. PLAG co-treatment promotes earlier expression of MIP-2 and endosome-related signals in LPS-stimulated Raw264.7 cells. Moreover, PLAG also induces a more rapid return of the TLR4 receptor to the plasma membrane. This suggests that PLAG effectively terminates endosome-dependent signaling and turns off MIP-2 expression in a shorter amount of time than in cells stimulated with LPS alone. Also, we confirmed that the use of inhibitors of CXCR2, a MIP-2 binding receptor, completely reduced neutrophil infiltration to the lung of mice with LPS treatment (data not shown). In this study, we focused that MIP-2 (CXCL2 or CXCL8) is a main chemoattractant for neutrophils. However, we also have confirmed that other LPS-induced chemokines such as CXCL5, CCL2, and CCL5 were reduced the mRNA expressions in BALF by PLAG co-treatment (Supplementary Figure 2). The reason is that LPS-induced ALI was promptly terminated by PLAG treatment. In other words, rapid resolution of acute inflammation by PLAG showed the reductions of other chemokines, its receptors, inflammatory cytokine and damage-associated molecular patterns expression in the lung of LPS-induced ALI mice. Generally, CCL2 and CCL5 are key chemokines that monocytes/macrophages to the site of inflammation (32). CXCL5 is well-known to chemotactic and activating functions on neutrophil and interacts with CXCR2 (33). CXCL5 is also known as epithelial-derived neutrophil activating peptide. Although we have checked only the mRNA expression of CXCL5 in BALF, it is possible that the decrease of CXCL5 expression also is regulated by PLAG. However, the reduction of CXCL5 mRNA expression after LPS/PLAG co-treatment was a small difference. Therefore, these data suggest that PLAG acts as pro-resolving molecule by limiting an excessive inflammatory response via effective control of MIP-2 expression. This is consistent with our previous reports, which showed PLAG efficacy in the control of inflammation. For example, PLAG improved survival in a mouse sepsis model, attenuated the infiltration of inflammatory cells into the airway in a mouse asthma model, and exerted an anti-tumor effect in a hamster biliary cancer model (34–36).

LPS stimulates TLR4-mediated endocytosis, and as noted above, PLAG induces an earlier internalization of TLR4 in cells treated with LPS. This internalization of TLR4 is also critical for induction of MIP-2 expression. Evidence for the role of PLAG in promoting endocytosis of TLR4 was further obtained by demonstrating the earlier recruitment of NOX subunits, p47phox and Rac1, as well as earlier ROS generation in cells co-treated with PLAG/LPS compared to LPS alone. Notably, we further found that PLAG only exerts an effect on the TLR4 TRIF-dependent endosomal-signaling pathway and not on the Myd88-dependent pathway (Figure 4). The rapid induction and fast termination of TRIF-dependent TLR4 signaling by PLAG suggests that this molecule can promote a faster return to homeostasis, thus avoiding unnecessary and harmful inflammatory responses. We propose that this may promote optimal ROS production for clearance of invading pathogens without excessive host cell damage.

In this study, we developed the ALI mouse model by directly and intranasally injecting 25 mg/kg of LPS into the mice. LPS is a typical endotoxin derived from gram negative bacteria, and have been widely utilized for nearly 100 years in bioresearch area because it replicates much of physiology of infection-associated disease including ALI, ARDS, and sepsis (37, 38). Despite its simplicity in learning the molecular mechanism, we admit the fact that the use of LPS has several inherent limitations. First, LPS is one of components of various pathogen associated molecular patterns (PAMPs) produced by gram-negative microorganisms (37, 38). Therefore, LPS models neglects host interaction to other gram-negative-derived PAMPs and infection to gram positive bacteria and poly-microbes (37, 38). Second, ALI/ALDS models can be developed via non-infectious methods such as acid aspiration, hyperoxia, surfactant depletion and lung ischemia reperfusion (39). In order to overcome these limitations, we investigated whether PLAG shows the therapeutic efficacy in acute pneumonia models by challenging both gram-negative and -positive bacteria, and PLAG effectively regulated neutrophil infiltration and clearance of invaded pathogens (data not shown). In previous study, PLAG also effectively suppressed cigarette smoke-induced neutrophilic inflammation in lung tissue by regulating pro-inflammatory cytokine production and ROS generation (40). Based on these observations, we are confident that PLAG would have a similar therapeutic efficacy in multiple and different ALI animal models.

**Figure 8:**
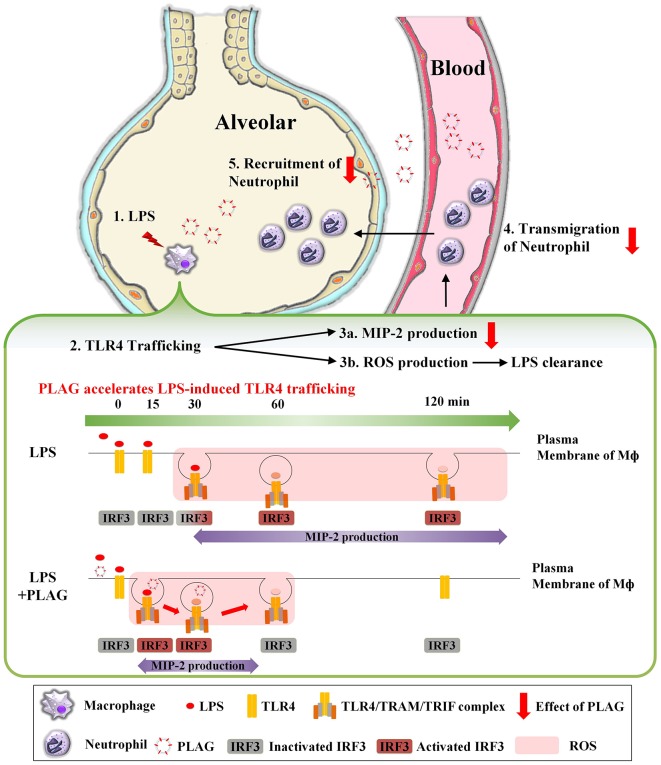
PLAG prevents ALI through reduction of neutrophil transmigration via acceleration of TLR4 trafficking. A serial cascade is expected to occur following LPS treatment, leading to the recruitment of neutrophils into the alveolar. (1) Exogenously injected LPS binds to MD2/TLR4 on the surface of macrophage in the alveoli, resulting in activation of macrophage. (2) Activated TLR4 binds to endocytosis-dependent adaptor protein such as TRAM and TRIF, and internalized into intracellular. Then, IRF3, which is a downstream of TLR4/TRAM/TRIF complex, is activated and acts as a transcription factor in the nucleus, (3a) expressing mRNA of MIP-2 and induces the production of MIP-2 protein. MIP-2 produced from macrophage forms a concentration gradient to recruit neutrophils into alveoli. (3b) Internalization of TLR4 also produces intracellular ROS. The generated ROS remove LPS and MIP-2 induces (4) transmigration and (5) recruitment of neutrophils into alveolar. Finally, the superfluous recruited neutrophils will damage to host tissue and occur the inflammation. At this time, PLAG treatment promotes trafficking of TLR4 that occurs in response to LPS on plasma membrane of macrophage. Therefore, PLAG induces rapid removal of LPS by promoting ROS generation time and induced rapid removal of ROS. In addition, the duration of activation of IRF3 by internalization of TLR4 is reduced, which ultimately reduces the total amount of MIP-2 expression and prevents the excessive recruitment of neutrophils to the alveolar space. Upon PLAG administration, LPS-induced neutrophil transmigration is attenuated via acceleration of TLR4 trafficking.

In summary, as shown in Figure 8, TLR4 on the surface of macrophage recognizes LPS injected into the alveolar space and internalized into cytosol. Internalization of TLR4 produces two responses. First, it induces TLR4/TRAM/TRIF/IRF3/MIP-2 signal cascade, an endocytosis dependent signaling pathway of TLR4. Second, ROS is generated to remove LPS. At this time, PLAG promotes LPS-induced TLR4 trafficking of macrophage surface. Therefore, by shortening the period of internalization of TLR4, it is possible to reduce the production of MIP-2, which acts as chemotaxis of neutrophils, to prevent excessive recruitment of neutrophils and to produce ROS in a shorter time, thereby promoting the clearance of LPS. Collectively, our data show that PLAG stimulates a more rapid resolution of LPS-induced lung inflammation, which suggests that it may hold potential as a therapeutic agent for various inflammatory diseases.

## Ethics Statement

All animal experimental procedures were performed in accordance with the Guide and Use of Laboratory Animals (Institute of Laboratory Animal Resources). All experiments were approved by the Institutional Review Committee for Animal Care and Use of KRIBB (Korea Research Institute of Bioscience and Biotechnology, Daejeon, Republic of Korea), approval number KRIBB-AEC-16031.

## Author Contributions

JWK and H-RL: conception and design. H-RL, S-HS, and JHK: development of methodology. H-RL, S-HS, and JHK: acquisition of data. JWK, H-RL, S-HS, JHK, and SY: analysis and interpretation of data. K-YS: supporting and provision of materials. JWK, H-RL, and SY: writing, review, and/or revision of the manuscript.

### Conflict of Interest Statement

H-RL, SY, and K-YS were employed by company, ENZYCHEM Lifesciences (Bio valley-ro, Jechon-si, Chungcheongbuk-do, Republic of Korea). The remaining authors declare that the research was conducted in the absence of any commercial or financial relationships that could be construed as a potential conflict of interest.
