# Supplementary material for: 1-Palmitoyl-2-Linoleoyl-3-Acetyl-rac-Glycerol (PLAG) Rapidly Resolves LPS-Induced Acute Lung Injury Through the Effective Control of Neutrophil Recruitment
Source: Front Immunol. 2019 Sep 18;10:2177. doi: 10.3389/fimmu.2019.02177 (PMC6759802; doi:10.3389/fimmu.2019.02177)
Supplement: Supplementary file 1 [file Data_Sheet_1.PDF]

Supplementary Figure 1.

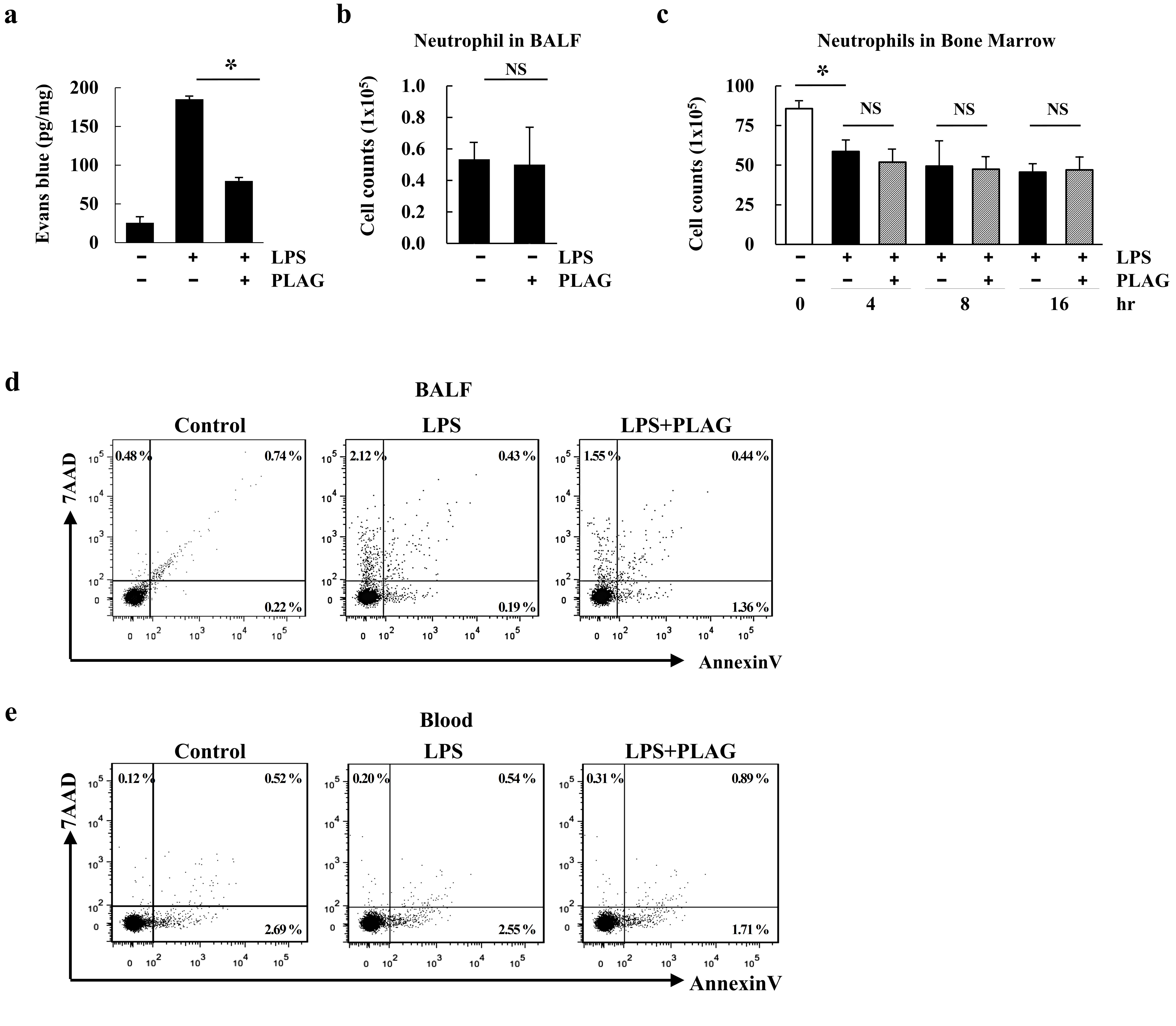

Supplementary Figure 2.

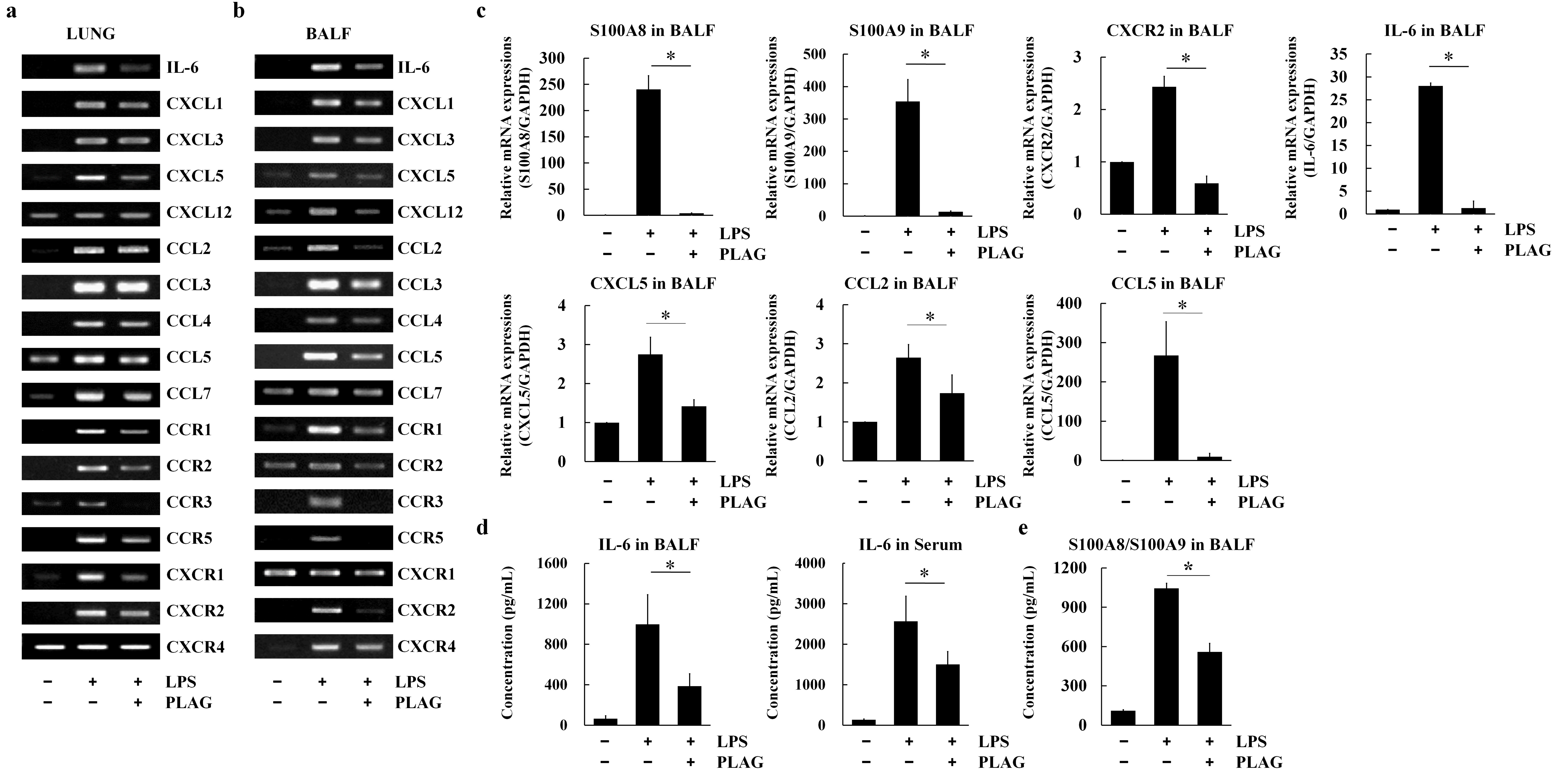

Supplementary Figure 3.

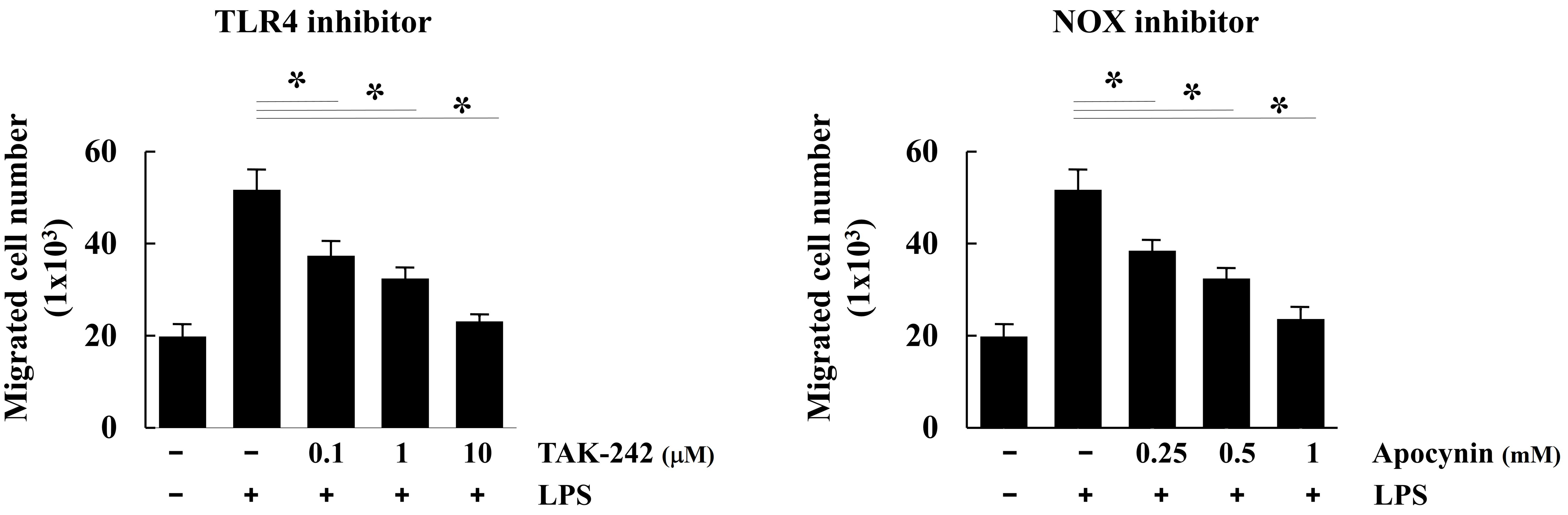

Supplementary Figure 4.

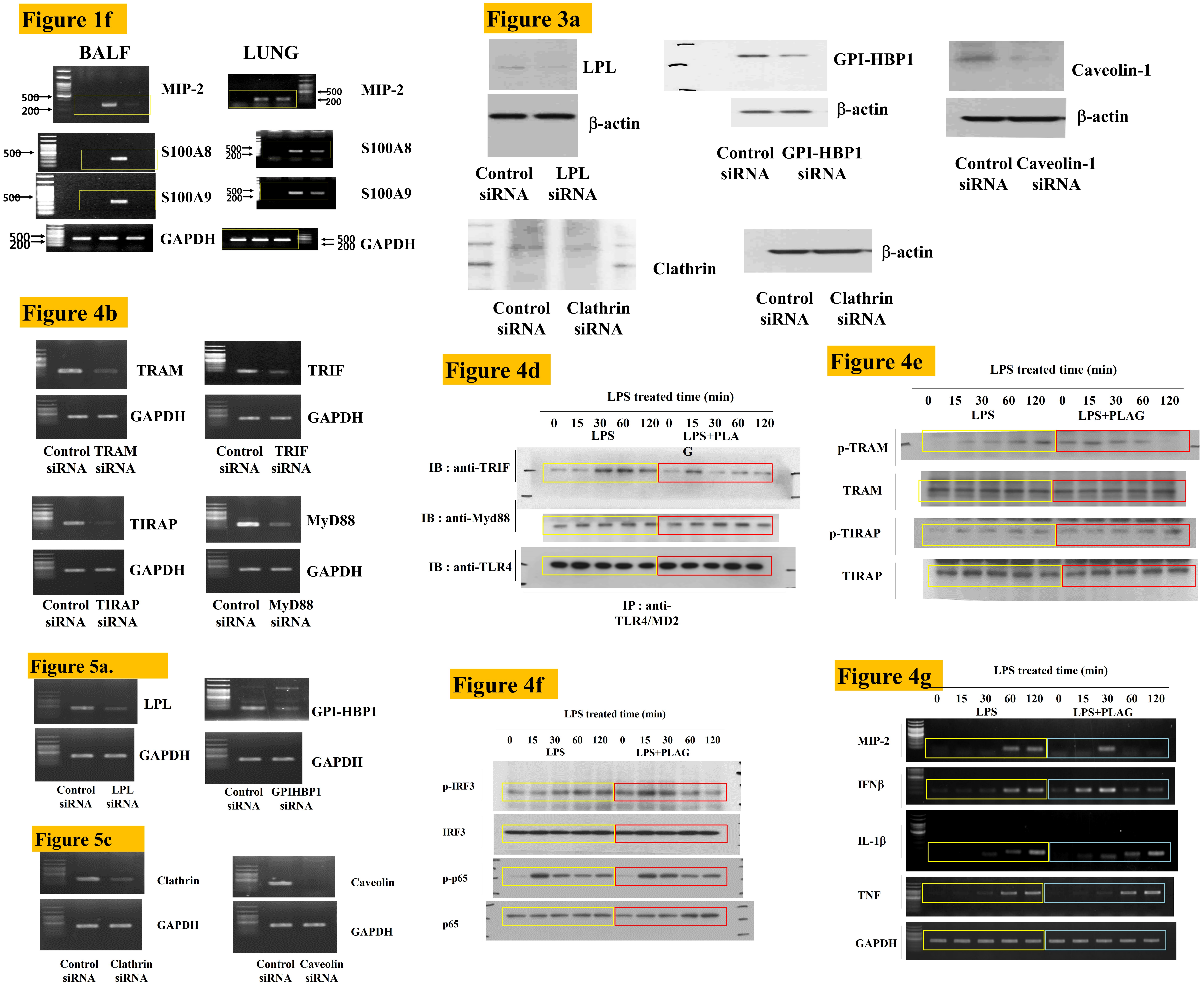

Supplementary Information

Supplementary Figure 1. PLAG reduces LPS-mediated vascular leakage in mouse lung tissue in vivo but alone has no effect on neutrophil migration, and PLAG/LPS co-treatment does not alter neutrophil release from bone marrow or apoptosis.

Mice were treated as described in Figure 1a. (a) Evans blue dye was extracted from lungs and quantified against standard curves as described in the Materials and Methods. (b) Mice were orally administrated with PLAG (250 mg/kg) alone, and the number of neutrophils in BALF was counted after 16 h. (c) Mice were treated as described in Figure 1e, and bone marrow-derived cells were harvested from femurs and tibias and counted after treatment for 4, 8, and 16 h. (d-e) Mice were treated with LPS and PLAG for 16 h; (d) BALF and (e) blood cells were stained with FITC-conjugated annexin V and 7-aminoactinomycin D to evaluate apoptosis. All experiments were performed in triplicate, and representative images are displayed. In vivo experiments were from at least three independent experiments with five mice for each group, and the bar represents the mean.* indicates $p<0.05$. NS, not significant.

Supplementary Figure 2. PLAG downregulates the expressions of pro-inflammatory cytokines and chemokines in the LPS-induced ALI model

Mice were treated as described in Figure 1a. Expression of various chemokines and cytokines in (a) Lung tissues and (b) BALF cells was analyzed using RT-PCR. (c) Expression of S100A8, S100A9, IL-6, CXCR2, CXCL5, CCL2, and CCL5 was measured in BALF cells by qPCR. GAPDH was used as a control. Primers used in this study were listed in Table 2. (d) Secreted levels of IL-6 in isolated BALF and serum were measured using an IL-6 ELISA assay. (e) Secreted level of S100A8/S100A9 heterodimer in isolated BALF was measured using an S100A8/S100A9 heterodimer ELISA assay. All Data represent one experiment performed in triplicate. * indicates $p<0.05$.

Supplementary Figure 3. Neutrophil transmigration is blocked by inhibitors of TLR4 and NOX.

Raw264.7 cells were incubated with TAK-242 (TLR4 inhibitor) or Apocynin (NOX inhibitor) for 1 h and stimulated with LPS (100 ng/ml) for 16 h. Cell supernatants were loaded into the lower chamber of a Transwell plate, and primary neutrophils from mouse bone marrow were accessed for their transmigration ability using Transwell assay described in the Materials and Methods. The migrated primary neutrophils were counted. All Data represent one experiment performed in triplicate. * indicates $p<0.05$.

Supplementary Figure 4. Uncropped gels and western blots.
